# Molecular Targets and Associated Signaling Pathways of Jingshu Granules in Ovarian Cysts Based on Systemic Pharmacological Analysis

**DOI:** 10.1155/2021/6660087

**Published:** 2021-01-21

**Authors:** Jili Xu, Yincong Xu, Ye Zhu, Zhihui Li, Xiaoping Wang

**Affiliations:** College of Traditional Chinese Medicine, Shandong University of Traditional Chinese Medicine, Jinan City, Shandong Province, 250355, China

## Abstract

**Background:**

More than a third of women could develop ovarian cysts during their lifetime. Jingshu granules are used for the treatment of gynecological disease of primary dysmenorrhea. However, the molecular mechanisms of Jingshu granules in ovarian cysts are still unreported. We aimed to find the active ingredients, molecular targets, and potential signaling pathways of Jingshu granules in ovarian cysts by using the systemic pharmacological analysis.

**Methods:**

Firstly, the effect of Jingshu granules on female hormones and reproductive organs of young female rats was evaluated. Secondly, candidate pharmaceutical ingredients of Jingshu granules were retrieved from the traditional Chinese medicine systems pharmacology (TCMSP) database and analysis platform. Potential protein targets for the active ingredients in Jingshu granules were then identified according to the oral bioavailability and drug-likeness indices. Thirdly, ovarian cyst-related gene targets were screened based on different databases. Finally, enrichment analysis was used to analyze the potential biological function of intersection targets between Jingshu granules and ovarian cysts.

**Results:**

In young female rats, Jingshu granules reduced the secretion of estradiol, progesterone, and prolactin and could affect the development of the uterus. This suggested that Jingshu granules played roles in hormone secretion and reproduction. From the TCMSP, a total of 1021 pharmaceutical ingredients of Jingshu granules were retrieved. After further screening, a total of 166 active ingredients and 159 protein targets of Jingshu granules were identified. In addition, 4488 gene targets of ovarian cysts were screened out. After taking the intersection, a total of 110 intersection targets were identified between potential protein targets of Jingshu granules and gene targets of ovarian cysts. In the functional analysis of 110 intersection targets, 8 signaling pathways including progesterone-mediated oocyte maturation (MAPK8 and CDK1 involved), GnRH signaling pathway (JUN involved), T cell receptor signaling pathway and Toll-like receptor signaling pathway (MAPK1 involved), NOD-like receptor signaling pathway (TNF, IL6, and IL1B involved), p53 signaling pathway (CDK2 and CDK4 involved), VEGF signaling pathway (MAPK14 involved), and PPAR signaling pathway (PPARG involved) were obtained.

**Conclusion:**

Our study revealed that Jingshu granules could function in patients with ovarian cysts through a number of molecular targets and signaling pathways. Our study may provide a new field into the mechanisms of Jingshu granules in ovarian cysts, from the molecular to the signaling pathway level.

## 1. Introduction

An ovarian cyst is a fluid-containing sac (>20 mm in diameter) that is formed in the ovaries. It is common in women of all ages, and its prevalence in pre- and postmenopausal women is 35% and 17%, respectively [1]. An ovarian cyst consists of five types, including follicular cysts, lutein cysts, cystic corpora lutea, cystic rete ovarii, and cysts of subsurface epithelial structures [2–4]. The most common symptoms of these cysts are dyspareunia, chronic pelvic pain, and gastrointestinal and urinary symptoms [5, 6]. Endometriosis, serous, mucinous, teratomas, inflammation, Brenner, and fibroma-thecoma are benign ovarian cysts, which result from the unruptured follicle. However, ovarian cysts (cysts larger than 40 mm and abnormal serum CA-125 levels) with intolerable symptoms may form the malignant lesion, such as the cystadenocarcinoma or cystadenoma [6, 7]. At this time, surgical removal of the cysts is required. However, the removal of ovarian cysts is also related to injury to the ovarian reserve [8, 9]. The pathology of ovarian cysts is complex. It is found that age, diet, the last menstrual period, chemotherapy, duration of tamoxifen, and serum levels of estrogen and follicle-stimulating hormone are associated with the occurrence of ovarian cysts [10, 11].

The traditional Chinese medicine (TCM) formula, composed of various active ingredients, has a complex action mechanism, which could be related to multiple targets and pathways. Recently, TCM has attracted remarkable attention in female reproductive dysfunctions in view of its safety and efficacy [12–16]. For example, Bushen Tianjing Recipe has been used for premature ovarian failure [17]. Jingshu granules consist of 11 herbs, including Angelica, radix Paeoniae alba, radix Bupleuri, nutgrass Galingale rhizome, radix Curcumae, rhizoma Ligustici wallichii, Corydalis tuber, Ginseng, rhizoma Atractylodis macrocephalae, dodder weed, and licorice. The water decoction of Jingshu granules has been in clinical application for more than 50 years and has a significant effect on the treatment of primary dysmenorrhea. However, few studies reported the action mechanism of Jingshu granules in other gynecological diseases, such as ovarian cysts. In this study, we tried to explore the action mechanisms of Jingshu granules, which could provide a basis for the understanding of Jingshu granules in the treatment of ovarian cysts.

## 2. Methods

### 2.1. The Effect of Jingshu Granules on Female Hormones and Reproductive Organs of Young Female Rats

In order to study the preliminary effect of Jingshu granules on female hormones and reproduction organs, 40 young SPF Sprague-Dawley (SD) female rats (sexually mature and unmated) aged 12 weeks old (240 ± 20 g) were selected in this study. These rats were purchased from Jinan Pengyue Experimental Animal Breeding Co., Ltd. (License No.: SCXK (Lu) 20180003). Animal experiments were conducted in the barrier environment facility of the Experimental Animal Center of Shandong University of Traditional Chinese Medicine (License Number: SYXK (Lu) 20170022) at room temperature of 23 ± 1°C and humidity at 45 ± 5% with the fluorescent light irradiation. These rats were fed with Co60 maintenance feed (feed production license no.: SCXK (Beijing) 20190003) and sterile water. They were randomly divided into four groups. In this study, the Jingshu granules are purchased from Shandong Hospital of Traditional Chinese Medicine. In the first group, the rats were orally given a high dose of Jingshu granules (15.2 g/kg), which was equivalent to 10 times the clinical dosage of the human clinical dose. In the second group, the rats were orally given a medium dose of Jingshu granules (7.6 g/kg), which was equivalent to 5 times the clinical dosage of the human clinical dose. The rats in the third group received a low dose of Jingshu granules (3.8 g/kg), which was equivalent to 2.5 times the human clinical dose. The rats in the fourth group were given an equal volume of water as the normal control. After continuous gavage for 30 days, 45 mg/kg sodium pentobarbital was injected intraperitoneally for anesthesia. Blood samples were collected from the inferior vena cava to measure progesterone, estradiol, and prolactin. The uterus and ovaries were dissected and weighed. All animal studies were approved by the Shandong University of Traditional Chinese Medicine (SDUTCM20181102001) and were in accordance with the Guidelines for Care and Use of Experimental Animals.

### 2.2. Identification of Active Ingredients and Protein Targets of Jingshu Granules

In order to explore the drug target of Jingshu granules, systemic pharmacological analysis was performed. All compounds of eleven herbs in Jingshu granules were retrieved from the TCM systems pharmacology (TCMSP) (http://lsp.nwu.edu.cn/tcmsp.php) database. The TCMSP includes information on all 500 TCM formulae [18]. Molecules with oral bioavailability (OB) ≥ 30% and drug-likeness (DL) ≥ 0.18 were considered active ingredients of Jingshu granules. The potential protein targets for the active ingredients in Jingshu granules were further identified by using the systematic drug targeting approach [19].

### 2.3. Identification of Ovarian Cyst-Related Gene Targets

In order to investigate the molecular mechanism of Jingshu granules in the hormones and reproduction-related diseases of ovarian cysts, the gene targets of ovarian cysts were retrieved from the therapeutic target database (TTD, http://db.idrblab.net/ttd/), DrugBank (https://www.drugbank.ca/), DisGeNET (https://www.disgenet.org/home/), Comparative Toxicogenomics Database (CTD, https://ctdbase.org/;jsessionid=0703B75089B9C2C2727D48FD74B43FA9), and MalaCards (https://www.malacards.org/). Only those intersection targets between potential protein targets for the active ingredients in Jingshu granules and gene targets of ovarian cysts were used for subsequent analysis.

### 2.4. Functional Analysis of Intersection Targets between Jingshu Granules and Ovarian Cysts

To further understand the potential biological function of intersection targets between Jingshu granules and ovarian cysts, Gene Ontology (GO) and Kyoto Encyclopedia of Genes and Genomes (KEGG) signaling pathway enrichment were performed via the DAVID database. The criterion for selecting significantly enriched functional terms of intersection targets was *p* < 0.05.

### 2.5. Statistical Analysis

Statistical calculation was performed using SPSS software (SPSS Inc., Chicago, IL, USA). Comparisons between two groups were assessed using Student's unpaired *t*-tests. A level of *p* < 0.05 was selected as the statistical significance in every comparison.

## 3. Results

### 3.1. The Effect of Jingshu Granules on Female Hormones and Reproductive Organs of Young Female Rats

From the result of Jingshu granules on female hormones of young female rats (Table 1), we found that the high, medium, and low doses of Jingshu granules significantly reduced the expression of prolactin. The high and medium doses of Jingshu granules remarkably reduced the expression of estradiol. In addition, the large dose group of Jingshu granules had a significant weakening effect on progesterone. This suggested that Jingshu granules could affect the secretion of female hormones. From the result of Jingshu granules on the reproductive organs of young female rats (Table 2), we found that the medium and low doses of Jingshu granules had no significant effect on the body weight and sexual organ weight of the young female rats. The high dose of Jingshu granules had no significant effect on the body weight and ovarian weight of the young female rats. However, a high dose of Jingshu granules could reduce the uterine weight of young female rats. This indicated that Jingshu granules could affect the development of the uterus.

### 3.2. Intersection Targets between Potential Protein Targets of Jingshu Granules and Gene Targets of Ovarian Cysts

**Figure 1:**
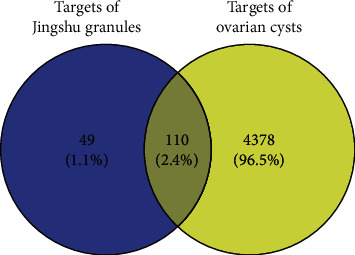
Venn diagram of targets between potential protein targets of Jingshu granules and gene targets of ovarian cysts.

Clinically, an ovarian cyst is a hormone- and reproduction-related disease. In view of the roles of Jingshu granules in hormone secretion and reproduction, we tried to investigate the molecular mechanism of Jingshu granules in ovarian cysts based on systemic pharmacological analysis. From the TCMSP, a total of 1021 pharmaceutical ingredients of Jingshu granules were retrieved. After further screening under the threshold of oral bioavailability (OB) ≥ 30% and drug-likeness (DL) ≥ 0.18, a total of 166 active ingredients and 159 protein targets of Jingshu granules were identified. In the TTD, DrugBank, DisGeNET, Comparative Toxicogenomics Database, and MalaCards, we found 4488 gene targets of ovarian cysts. After taking the intersection, a total of 110 intersection targets between potential protein targets of Jingshu granules and gene targets of ovarian cysts were identified (Figure 1). These 110 intersection targets were targeted by 165 active ingredients in 11 herbs of Jingshu granules. 165 active ingredients were from 9 in radix Paeoniae alba, 4 in rhizoma Atractylodis macrocephalae, 14 in radix Bupleuri, 7 in rhizoma Ligustici wallichii, 2 in Angelica, 58 in licorice, 19 in Ginseng, 9 in dodder weed, 17 in nutgrass Galingale rhizome, 49 in Corydalis tuber, and 4 in radix Curcumae. Among which, 10 active ingredients were found in two or more herbs.

### 3.3. Functional Analysis of Intersection Targets between Jingshu Granules and Ovarian Cysts

**Figure 2:**
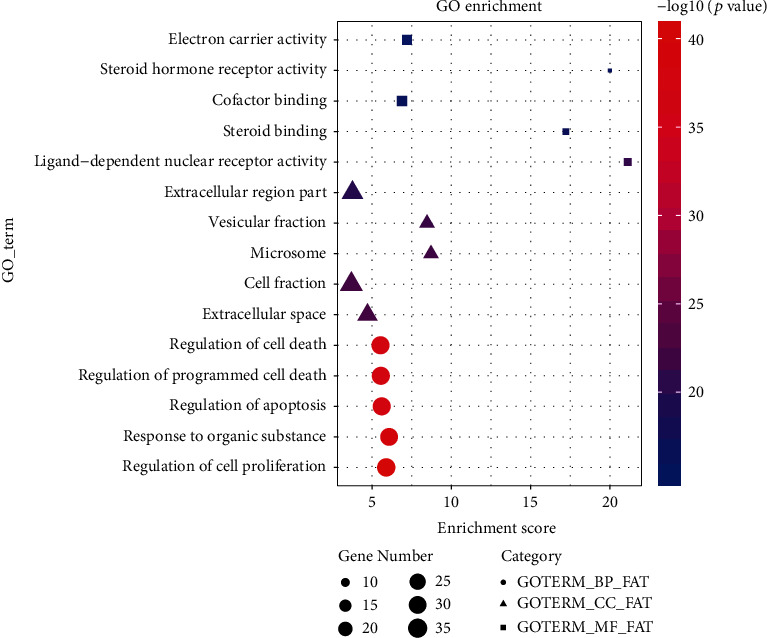
Top 15 enriched GO terms of intersection targets between Jingshu granules and ovarian cysts.

**Figure 3:**
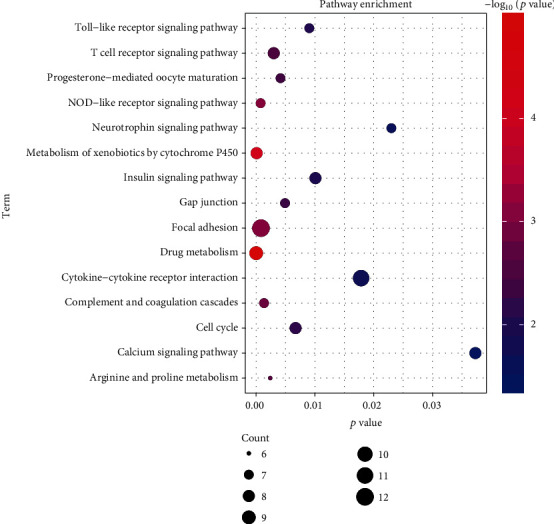
Top 15 enriched KEGG signaling pathways of intersection targets between Jingshu granules and ovarian cysts.

**Figure 4:**
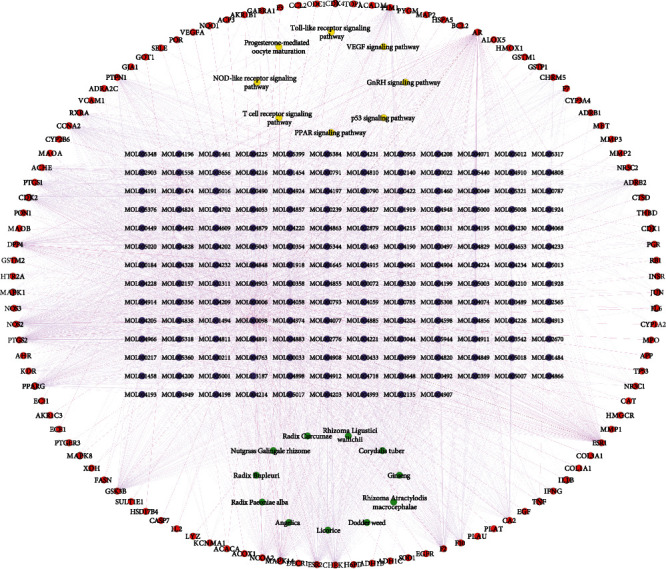
The network with 11 herbs, 165 active ingredients, 110 intersection targets, and 8 signaling pathways. Green circle, purple circle, red circle, and yellow circle represented the herb, active ingredient, intersection target, and signaling pathway, respectively. Black circle line represented 11 intersection targets in 8 signaling pathways.

In order to understand the potential biological function of intersection targets between Jingshu granules and ovarian cysts, GO and KEGG signaling pathway enrichment analysis was performed. The top 15 enriched GO terms and KEGG pathways are shown in Figures 2 and 3, respectively. In addition, all KEGG signaling pathways and involved genes are listed in Table 3 in detail. Among which, 8 signaling pathways were identified, such as progesterone-mediated oocyte maturation (MAPK8 and CDK1 involved), GnRH signaling pathway (JUN involved), T cell receptor signaling pathway and Toll-like receptor signaling pathway (MAPK1 involved), NOD-like receptor signaling pathway (TNF, IL6, and IL1B involved), p53 signaling pathway (CDK2 and CDK4 involved), VEGF signaling pathway (MAPK14 involved), and PPAR signaling pathway (PPARG involved). Information of the above 11 involved ovarian cyst-related targets of Jingshu granules is shown in Table 4. Additionally, the network with 11 herbs, 165 active ingredients, 110 intersection targets, and 8 signaling pathways is shown in Figure 4.

## 4. Discussion

Some functional cysts, including cystic follicles and follicular cysts, contain estrogen in the luminal fluid [20]. The detailed mechanism underlying the cystic changes in the ovary remains unknown, although the relationship between ovarian cysts and increased estrogen levels has been found [5]. Knauf et al. found the increased progesterone concentrations in cyst fluid [6]. Prolactin metabolism disorder has been considered one of the pathological mechanisms of multicystic ovarian disease [21]. Perreault et al. proposed that suppression of prolactin secretion could solve some gynecologic problems [22]. Thus, it can be seen that estrogen, increased levels of progesterone, and prolactin are associated with the ovarian cyst. In this study, we found that Jingshu granules could affect the secretion of estradiol, progesterone, and prolactin and affect the development of the uterus of young female rats. This suggested that Jingshu granules played roles in hormone secretion and reproduction, which may be a potential drug for ovarian cyst treatment.

In order to investigate the molecular mechanism of Jingshu granules in the hormones and reproduction-related diseases of ovarian cysts, systemic pharmacological analysis was performed to find active ingredients and protein targets of Jingshu granules and ovarian cyst-related gene targets. Significantly, 110 intersection targets were identified between potential protein targets of Jingshu granules and gene targets of ovarian cysts. According to the KEGG enrichment analysis of 110 intersection targets, 8 signaling pathways including progesterone-mediated oocyte maturation (MAPK8 and CDK1 involved), GnRH signaling pathway (JUN involved), T cell receptor signaling pathway and Toll-like receptor signaling pathway (MAPK1 involved), NOD-like receptor signaling pathway (TNF, IL6, and IL1B involved), p53 signaling pathway (CDK2 and CDK4 involved), VEGF signaling pathway (MAPK14 involved), and PPAR signaling pathway (PPARG involved) were obtained.

Progesterone could enhance the production of proteolytic enzymes that are important for the follicle rupture at ovulation [23]. In polycystic ovary syndrome, some miRNAs target a number of genes to play different functions, such as progesterone-mediated oocyte maturation [24]. Upregulation of MAPK8 (an apoptosis-related gene) is related to oocyte dysfunction and is involved in various signaling pathways in granulosa cells [25–34]. It is speculated that MAPK8 is one of the therapeutic targets for polycystic ovary syndrome [35]. The CDK1 is an important signaling pathway that is evolutionarily conserved in controlling oocyte growth and meiotic maturation. Kanatsu-Shinohara et al. have demonstrated that CDK1 is an important limiting factor in female gametes [36]. The expression of CDK1 is increased in mature oocytes [37]. In rat oocytes, sustained reduced levels of Thr-161-phosphorylated CDK1 destabilize the maturation-promoting factor [38, 39]. In addition, CDK1-null oocytes are permanently arrested at the GV stage, suggesting that CDK1 activity is crucial to the resumption of the first meiosis [40]. Our result indicated that Jingshu granules may exert a medicinal effect by targeting the MAPK8 and CDK1 of the progesterone-mediated oocyte maturation signaling pathway in ovarian cysts.

It is reported that slow GnRH pulse frequencies favor follicle-stimulating hormone production and secretion, whereas rapid frequencies favor luteinizing hormone production and secretion [41–43]. It is worth mentioning that GnRH-a is related to the formation of functional ovarian cysts [44]. In the mature gonadotrope cells, GnRH also induces c-JUN transcription, which leads to upregulation of cell cycle proteins [45]. In granulose cells, the conjunction of c-JUN and TAF4b regulates the expression of ovarian genes [46]. In the follicle cells, knockdown of JUN results in engulfment defects in the ovary [47]. It is noted that JUN has been identified as a potential key regulator in the development of polycystic ovary syndrome. Furthermore, decreased levels of p-c-JUN have been found in the ovary tissues of polycystic ovary syndrome rats [48]. Thus, it can be seen that JUN in the GnRH signaling pathway may play an important regulatory role in the ovary. Jingshu granules could be used for ovarian cyst treatment by targeting JUN in the GnRH signaling pathway.

Immune system-related cells (T cells) influence the differentiation of primordial follicles and the formation of granulosa cells [49–51]. It has been demonstrated that the absence of regulatory T cells is a prerequisite for cyst formation in the polycystic ovary syndrome [52]. The expression of innate immune system-related Toll-like receptors 2 and 4 was found in the ovarian cumulus cells and granulosa cells during ovulation [53]. It is found that other Toll-like receptor types are found in regulatory T cells from patients with endometriosis [54]. MAPK1 is involved in regulating cell proliferation and apoptosis [55, 56]. The signaling pathway of MEK/MAPK1/3 is crucial to oocyte complex expansion and follicle rupture [57–59]. It is found that MAPK1 is differentially expressed in the endometrium in ovarian endometriosis [60]. In the ovarian tissue of polycystic ovary syndrome patients, MAPK1 could activate autophagy [61]. It is noted that increased expression of MAPK1 had been found in the ovarian cystic epithelium [62]. Herein, we found that MAPK1 was involved in both the T cell receptor signaling pathway and the Toll-like receptor signaling pathway. This suggested the role of these signaling pathways in the therapy of ovarian cysts by Jingshu granules.

It is reported that the NOD-like receptor signaling pathway is highly involved in the development of polycystic ovary syndrome [63]. In polycystic ovary syndrome, hypermethylation in TNF contributes to androgen excess [64]. Moreover, TNF suppresses follicle-stimulating hormone-induced activation of the chorionic gonadotropin receptor promoter, making it a key factor contributing to hyperandrogenemia [65]. Interestingly, the expression of TNF-*α* has been found in serum and peritoneal fluid of women with benign serous ovarian cysts [66]. Brewer and Balen found that IL6 could stimulate the expansion of the cumulus-oocyte complexes and affect oocyte maturation through alterations in steroidogenesis and interaction with metabolic hormones [67, 68]. It is inferred that the single nucleotide polymorphism in IL6 is a genetic cause of polycystic ovary syndrome [69–71]. IL1B is involved in the regulation of ovarian primordial follicle assembly [72]. It is hypothesized that high expression of IL1B in granulosa cells induces a premature influx of leukocytes and impairs maturation and subsequent ovulation [73]. In the present study, we found that TNF, IL6, and IL1B were all involved in the NOD-like receptor signaling pathway, which may provide a new field for Jingshu granules in the treatment of ovarian cysts.

The presence of a mutation in the gene p53 is common in type II ovarian cancer [66]. CDK2, highly expressed in the ovary libraries, plays a crucial role in controlling follicle cell cycles [74, 75]. Satyanarayana and Kaldis found that the knockout of CDK2 destroyed the fertility of female mice [76]. CDK4 is associated with follicular growth [77]. In addition, CDK4 is critical for luteal function and granulosa cell proliferation [78]. In this study, we found that both CDK2 and CDK4 were involved in the p53 signaling pathway. This indicated that Jingshu granules may affect the follicle cell cycle in the development of ovarian cysts.

The increased expression of VEGF in the theca layer of the ovary leads to a decrease in ovulation [79–81]. In polycystic ovary syndrome, elevated VEGF prevents granulosa cell apoptosis and follicle atresia, which contribute to the growth and persistence of follicles [82]. However, overexpression of VEGF receptors could be related to the accumulation of follicular fluid and abnormal follicular growth. In addition, VEGF is involved in other reproduction disorders, such as endometriosis [83]. It is found that the luteinizing hormone surge could trigger follicular maturation and ovulation by activating the MAPK14 signaling pathway in granulosa cells [84, 85]. In addition, MAPK14 plays a role in androgen biosynthesis [86]. The expression of MAPK14 has been found in patients with endometriosis and polycystic ovary syndrome [86, 87]. Our result suggested that Jingshu granules may promote ovulation by targeting MAPK14 in the VEGF signaling pathway in the therapy of ovarian cysts.

PPAR*γ*, a subtype of PPAR, is an important transcription factor associated with follicular differentiation [88]. Downregulation of PPAR*γ* in response to the luteinizing hormone is crucial to ovulation and luteinization [89]. PPARG, regulated by the progesterone receptor, is crucial to follicular maturation and remodeling [90]. In granulosa cells, PPARG is involved in nuclear signaling receptors [91]. The genetic association between PPARG and endometriosis has been demonstrated [92]. Our study indicated that PPARG in the PPAR signaling pathway may be under the regulation of Jingshu granules for ovarian cyst treatment.

## 5. Conclusion

Our study reported multiactive ingredients in therapeutics by Jingshu granules to provide multitarget, multisignaling pathway regulation of ovarian cyst activity. The mechanism of action of Jingshu granules in ovarian cysts involved multiple active ingredients, targets, and signaling pathways. The systemic pharmacological analysis may provide a comprehensive understanding of the mechanisms of Jingshu granules in ovarian cysts. However, there is a limitation to our study. In vivo or in vitro experiments are lacking. Experimental verification of identified potential targets and signaling pathways of Jingshu granules in ovarian cysts is needed.

## Figures and Tables

**Table 1:** The effect of Jingshu granules on female hormones of young female rats.

Group	Dose (g/kg)	Estradiol (pg/ml)	Progesterone (ng/ml)	Prolactin (ng/ml)
High	15.2	27.8 ± 15.2^∗∗^	0.58 ± 0.16^∗∗^	2.45 ± 1.28^∗∗^
Medium	7.6	30.3 ± 8.9^∗^	0.74 ± 0.20	5.27 ± 1.62^∗∗^
Low	3.8	37.5 ± 8.2	0.94 ± 0.85	6.63 ± 2.84^∗∗^
Normal control	Equal volume of water	42.9 ± 11.7	0.92 ± 0.24	9.62 ± 1.29

The result was represented as mean ± SD. ^∗^*p* < 0.05, ^∗∗^*p* < 0.01 compared with the normal control.

**Table 2:** The effect of Jingshu granules on the reproductive organs of young female rats.

Group	Dose (g/kg)	Body weight (g)	Uterus coefficient (mg/100 g)	Ovary coefficient (mg/100 g)
High	15.2	180.0 ± 15.7	28.6 ± 6.4^∗^	35.7 ± 7.9
Medium	7.6	186.6 ± 10.5	32.2 ± 8.0	36.8 ± 5.0
Low	3.8	185.2 ± 15.9	39.9 ± 9.2	45.0 ± 7.9
Normal control	Equal volume of water	193.3 ± 21.6	39.3 ± 10.4	39.8 ± 5.6

The result was represented as mean ± SD. ^∗^*p* < 0.05 compared with the normal control.

**Table 3:** All enriched KEGG signaling pathways of intersection targets between Jingshu granules and ovarian cysts.

Term	*p* value	Genes
Neuroactive ligand-receptor interaction	1.75*E* − 07	OPRM1, DRD1, DRD2, GABRA5, NR3C1, CHRM5, HTR1B, ADRB2, ADRB1, CHRM4, CHRM3, GRIA2, CHRM2, CHRM1, F2, ADRA1B, ADRA1A, ADRA2C, ADRA2B, ADRA1D, HTR2A
Calcium signaling pathway	2.47*E* − 06	EGFR, DRD1, CHRM5, ADRB2, ADRB1, CHRM3, CHRM2, CHRM1, ADRA1B, ADRA1A, NOS3, PRKACA, NOS2, PPP3CA, ADRA1D, HTR2A
Focal adhesion	0.002679	EGFR, MAPK1, JUN, GSK3B, BCL2, MET, VEGFA, COL3A1, MAPK8, COL1A1, EGF, KDR
Cytokine-cytokine receptor interaction	0.04291	EGFR, IL6, TNF, CCL2, MET, VEGFA, IFNG, IL1B, EGF, KDR, IL2
MAPK signaling pathway	0.047776	EGFR, MAPK1, TNF, MAPK14, JUN, TP53, IL1B, MAPK8, PRKACA, PPP3CA, EGF
Gap junction	7.78*E* − 05	EGFR, CDK1, MAPK1, DRD1, ADRB1, DRD2, GJA1, PRKACA, EGF, HTR2A
T cell receptor signaling pathway	0.001614	MAPK1, TNF, MAPK14, JUN, GSK3B, IFNG, PPP3CA, CDK4, IL2
NOD-like receptor signaling pathway	2.53*E* − 04	MAPK1, IL6, TNF, CCL2, MAPK14, IL1B, MAPK8, CASP1
Progesterone-mediated oocyte maturation	0.001846	PGR, CDK1, MAPK1, MAPK14, MAPK8, PRKACA, CCNA2, CDK2
Cell cycle	0.014242	CDK1, GSK3B, TP53, CHEK1, RB1, CDK4, CCNA2, CDK2
Drug metabolism	0.001617	GSTM1, CYP3A4, CYP2B6, MAOA, MAOB, CYP1A2, GSTP1
Complement and coagulation cascades	0.002808	PLAT, F10, THBD, F3, F2, F7, PLAU
VEGF signaling pathway	0.004269	MAPK1, PTGS2, MAPK14, VEGFA, NOS3, PPP3CA, KDR
Apoptosis	0.008778	TNF, BCL2, CASP7, TP53, IL1B, PRKACA, PPP3CA
GnRH signaling pathway	0.015262	EGFR, MAPK1, MAPK14, JUN, MAPK8, PRKACA, MMP2
Toll-like receptor signaling pathway	0.017491	MAPK1, IL6, TNF, MAPK14, JUN, IL1B, MAPK8
Oocyte meiosis	0.025501	PGR, CDK1, MAPK1, AR, PRKACA, PPP3CA, CDK2
Neurotrophin signaling pathway	0.042293	MAPK1, MAPK14, JUN, GSK3B, BCL2, TP53, MAPK8
Arginine and proline metabolism	0.004458	ODC1, GOT1, MAOA, MAOB, NOS3, NOS2
Arachidonic acid metabolism	0.005652	AKR1C3, PTGS2, CYP2B6, PTGS1, LTA4H, ALOX5
Metabolism of xenobiotics by cytochrome P450	0.007574	GSTM1, AKR1C3, CYP3A4, CYP2B6, CYP1A2, GSTP1
Glioma	0.009285	EGFR, MAPK1, TP53, RB1, EGF, CDK4
ErbB signaling pathway	0.033168	EGFR, MAPK1, JUN, GSK3B, MAPK8, EGF
p53 signaling pathway	0.049093	CDK1, TP53, CHEK1, CDK4, CDK2
Tryptophan metabolism	0.048697	MAOA, MAOB, CYP1A2, CAT

**Table 4:** Information regarding ovarian cyst-related targets of Jingshu granules.

Target_ID	Target_name	Gene
TAR00431	Cell division control protein 2 homolog	CDK1
TAR00482	Cell division protein kinase 2	CDK2
TAR00573	Cell division protein kinase 4	CDK4
TAR00418	Interleukin-1 beta	IL1B
TAR00351	Interleukin-6	IL6
TAR00414	Transcription factor AP-1	JUN
TAR00354	Mitogen-activated protein kinase 1	MAPK1
TAR00402	Mitogen-activated protein kinase 14	MAPK14
TAR00704	Mitogen-activated protein kinase 8	MAPK8
TAR00078	Peroxisome proliferator-activated receptor gamma	PPARG
TAR00265	Tumor necrosis factor	TNF

## Data Availability

All data are available in the article.
